# Cholecalciferol Additively Reduces Serum Parathyroid Hormone Levels in Severe Secondary Hyperparathyroidism Treated with Calcitriol and Cinacalcet among Hemodialysis Patients

**DOI:** 10.3390/nu10020196

**Published:** 2018-02-10

**Authors:** Cai-Mei Zheng, Chia-Chao Wu, Chi-Feng Hung, Min-Tser Liao, Jia-Fwu Shyu, Yung-Ho Hsu, Chien-Lin Lu, Yuan-Hung Wang, Jing-Quan Zheng, Tian-Jong Chang, Yuh-Feng Lin, Kuo-Cheng Lu

**Affiliations:** 1 Graduate Institute of Clinical Medicine, College of Medicine, Taipei Medical University, Taipei 11031, Taiwan; 11044@s.tmu.edu.tw (C.-M.Z.); yhhsu@s.tmu.edu.tw (Y.-H.H.); janlin0123@gmail.com (C.-L.L.); 12072@s.tmu.edu.tw (Y.-H.W.); jingquan235@gmail.com (J.-Q.Z.); 2 Division of Nephrology, Department of Internal Medicine, Shuang Ho Hospital, Taipei Medical University, New Taipei City 235, Taiwan; 3 Department of Internal Medicine, School of Medicine, College of Medicine, Taipei Medical University, Taipei 11031, Taiwan; 4 Division of Nephrology, Department of Internal Medicine, Tri-Service General Hospital, National Defense Medical Center, Taipei 114, Taiwan; wucc@ndmctsgh.edu.tw; 5 School of Medicine, Fu-Jen Catholic University, New Taipei City 242, Taiwan; 054317@gmail.com; 6 Graduate Institute of Biomedical and Pharmaceutical Science, Fu-Jen Catholic University, New Taipei City 242, Taiwan; 7 Department of Pediatrics, Taoyuan Armed Forces General Hospital, Taoyuan 325, Taiwan; liaoped804h@yahoo.com.tw; 8 Division of Pediatrics, Department of Medicine, Tri-Service General Hospital, National Defense Medical Center, Taipei 114, Taiwan; 9 Department of Biology and Anatomy, National Defense Medical Center, Taipei 114, Taiwan; shyujeff@mail.ndmctsgh.edu.tw; 10 Division of Nephrology, Department of Medicine, Cardinal-Tien Hospital & Fu-Jen Catholic University Hospital, School of Medicine, Fu-Jen Catholic University, New Taipei City 242, Taiwan; 11 Department of Medical Research, Shuang Ho Hospital, Taipei Medical University, New Taipei City 235, Taiwan; 12 Division of Critical Care, Department of Critical Care Medicine, Shuang Ho Hospital, Taipei Medical University, New Taipei City 235, Taiwan; 13 Graduate Institute of Life Sciences, National Defense Medical Center, Taipei 114, Taiwan; 10247@s.tmu.edu.tw; 14 Performance Appraisal Section, Secretary Office, Shuang Ho Hospital, Taipei Medical University, Taipei 235, Taiwan

**Keywords:** cholecalciferol, hemodialysis, secondary hyperparathyroidism, cinacalcet, calcitriol

## Abstract

We evaluated the improvement of intact parathyroid hormone (iPTH) levels and bone parameters by supplementing nutritional vitamin D (cholecalciferol) to combined calcimimetic (cinacalcet) and active vitamin D analog (calcitriol) among severe secondary hyperparathyroidism (SHPT) hemodialysis (HD) patients. A randomized, controlled open-label study was undertaken in 60 HD patients with serum iPTH > 1000 pg/mL or persistently high iPTH ≥ 600 pg/mL even after >3 months of calcitriol (3 μg/week). The study group received oral cholecalciferol (5000 IU/ day) and the control group received a placebo. All patients received fixed dose cinacalcet (30 mg/day, orally) and calcitriol. Calcitriol was reduced if iPTH ≤ 300 pg/mL and cinacalcet was withdrawn if serum iPTH was persistently low (iPTH ≤ 300 pg/mL) for 4 weeks after the reduction of calcitriol. A significantly lower iPTH level was noted from the 20th week in the study group compared to the placebo group, and the target iPTH ≤ 300 pg/mL was achieved at the 24th week in the study group. Most patients achieved serum 25-(OH)D_3_ ≥ 30 ng/mL in the study group. Nearly 40% of study patients gained >10% improvement in femoral neck (FN) bone mineral density (BMD). We conclude that cholecalciferol additively reduced serum iPTH levels, improved 25-(OH)D_3_ levels and improved FN BMD when used together with cinacalcet/calcitriol in severe SHPT HD patients.

## 1. Introduction

Secondary hyperparathyroidism (SHPT) is a major complication among dialysis patients which can have renal osteodystrophy and cardiovascular consequences [1,2]. Dietary control, phosphate binders and active vitamin D analogs are used in earlier SHPT whereas calcimimetic agent, cinacalcet, is indicated in later stages of SHPT cases who have markedly elevated parathyroid hormone (PTH) levels or failed to respond earlier treatment. The doses of active vitamin D analogs are reduced or cinacalcet is added when the patients have progressively high calcium, phosphate and Ca × P products to prevent cardiovascular and soft-tissue calcification [3,4]. Many studies demonstrated that cinacalcet improves PTH control and achieves recommended serum calcium and phosphorus values when used in combination with active vitamin D analogs and phosphate binders [5,6,7,8,9]. In our previous study, we revealed additional PTH lowering and anti-inflammatory effects of nutritional vitamin D (cholecalciferol) supplementation to active vitamin D analogs in SHPT patients [10]. However, data regarding the addition of cholecalciferol supplementation to combined cinacalcet and active vitamin D analogs in severe SHPT patients is still lacking.

Circulating PTH levels were found to be inversely correlated with serum 25(OH) D_3_, a circulating vitamin D metabolite which indicated the vitamin D status [11,12]. 25(OH)D_3_ was an important substrate for the local generation of 1,25(OH)_2_D with the help of local 1α-hydroxylase (1α-OHase) activity [13,14]. Most end-stage renal disease (ESRD) patients had low serum 25(OH)D_3_ levels [15,16]. Segersten et al. [17] revealed 1α-OHase expression in parathyroid glands which presumably suppressed the PTH gland hyperplasia in an autocrine/paracrine manner. Previous research revealed coincident increased expression of 1α-OHase (approximately increased in 10 folds) and reduced 24-hydroxylase in most SHPT glands [18] and highlighted the requirement of more 25(OH)D_3_ in these patients. CS Ritter et al. [19] proved that the local effect of 25(OH)D_3_ on PTH suppression possibly occurs through direct activation of the vitamin D receptor (VDR) in parathyroid glands. Furthermore, 25(OH)D_3_ played less role in systemic hypercalcemia and related complications. These findings explain the possible additive role of nutritional vitamin D (cholecalciferol) supplementation in SHPT patients.

Among two types of parathyroid cells (chief cells (CC) and oxyphil cells (OC)), OC markedly increased in chronic kidney disease (CKD) [20]. Lomonte C et al. revealed calcitriol therapy significantly increases the OC content in parathyroid glands [21]. Cinacalcet acts through CaR in the CC of parathyroid glands and exerts antiproliferative and proapoptotic action [22,23]. Studies revealed that cinacalcet significantly increases the OC/CC ratio (approx. increase 3.42 times) [24] and increases the oxyphil area [25]. These OC excessively express 1α OHase enzyme and increase local calcitriol production [26], which further carries out autocrine/paracrine regulation of PTH synthesis and release. Thus, cinacalcet use further increases the requirement of substrate chocalciferol for 1α OHase to produce local calcitriol production in OC. Calcimimetics also up-regulate decreased parathyroid CaR and VDR in both in vitro and in vivo studies [27,28] which further mediates parathyroid proliferation.

Therefore, we speculate that combining cinacalcet to calcitriol therapy increases the parathyroid OC/CC ratio, increases local 1αOHase activity, and increases VDR expression which additively needs more 25(OH)D_3_ for local calcitriol synthesis. We hypothesize that the cholecalciferol supplementation in SHPT patients together with calcitriol and cinacalcet therapy increase local calcitriol production, which further suppresses intact parathyroid hormone (iPTH) secretion. We further describe the changes in bone turnover markers and bone densities with or without cholecalciferol supplementation in SHPT hemodialysis patients.

## 2. Materials and Methods

### 2.1. Study Design

The trial was designed as a randomized, controlled open-label study. A total of 80 patients were eligible and agreed to participate. Group matching with gender, age (within 5 years), and duration of hemodialysis (within 1 year) was conducted for every pair group and they were then randomly assigned to either treatment with cinacalcet, calcitriol and cholecalciferol (CCC, study group) or control group, treated with cinacalcet, calcitriol and placebo (CCP, control group). G*power was used to calculate the required sample size [29] and effects were detected in a two-sided test with a power of (1 − β) = 80% at a significance level of 0.05. Other calculation settings were as follows: (1) the randomization process was based on 1:1 proportion of this study; (2) the effect size was set as 0.8. The required sample size for calculating was at least 25 subjects in the CCC group and 25 subjects in the CCP group.

### 2.2. Patient Eligibility and Randomization

**Figure 1:**
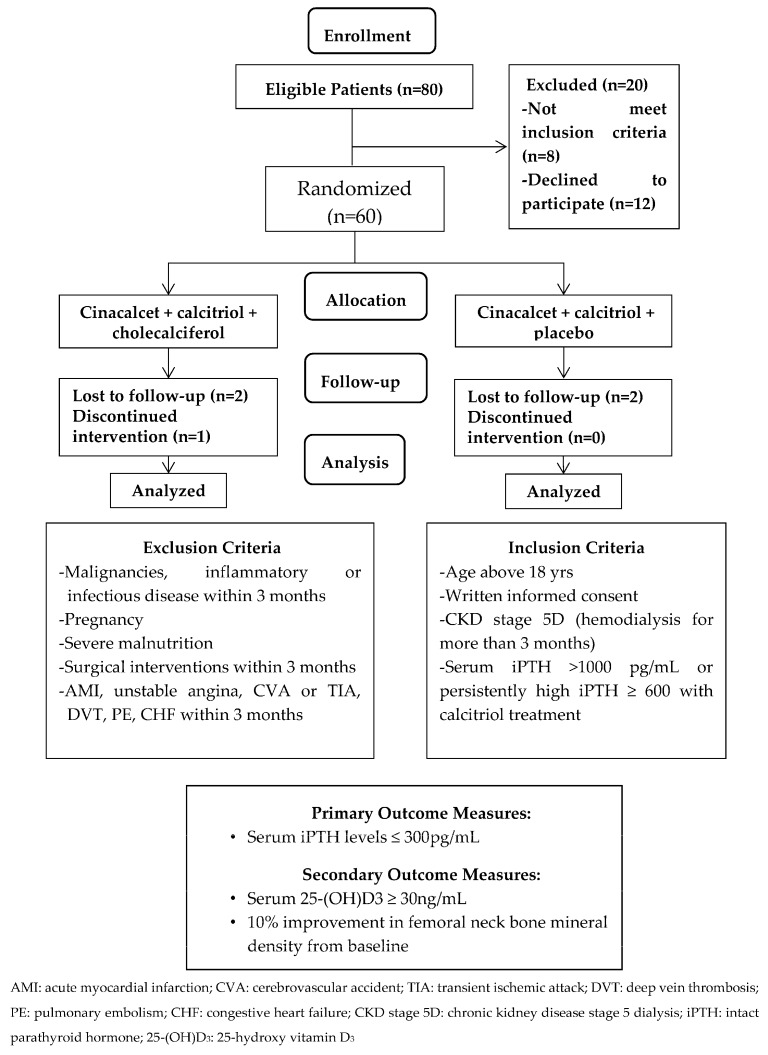
Study participants’ flow chart.

Patients aged above 18 years treated with maintenance hemodialysis three times per week for at least 3 months before screening were eligible. Patients with severe SHPT (serum iPTH > 1000 pg/mL or persistantly high serum iPTH ≥ 600 pg/mL even after more than 3 months of calcitriol treatment) were enrolled. All patients needed to stop receiving an active vitamin D at least 30 days before entering the study. Patients using calcimimetic agents and/or native vitamin D analogs were excluded. The patients were excluded if they were pregnant, breastfeeding or of childbearing potential and not practicing birth control. Those with malignancies, severe malnutrition, and inflammatory or infectious disorders diagnosed for more than 3 months before the study were also excluded. Other exclusion criteria included surgical interventions and vascular diseases, including acute coronary syndrome, unstable angina, cerebrovascular accident, transient ischemic attack, deep vein thrombosis, pulmonary embolism, or congestive heart failure within 3 months of the study period. The patients were also excluded if they had a history of allergy to medications (Figure 1). Sixty hemodialysis (HD) patients fulfilled the criteria and were randomized into the CCC study group (*N* = 30) and CCP control group (*N* = 30). Patients were well matched by treatment allocation (Figure 1). Protocol and informed consent were approved by the authorities of the Institutional Review Board of Cardinal Tien Hospital and Taipei Medical University (CTH106A-2B01 and CTH-104-3-5-022). All the patients provided written consent before study enrollment.

### 2.3. Treatment Intervention

All patients were given oral cinacalcet (30 mg/day) from the start of the study. Patients in the study group were given an oral form of cholecalciferol 5000 IU per day (Healthy Origens, Pittsburgh, PA 15241, USA); and cholecalciferol placebo (olive oil) was given in the control group. During the treatment, the calcitriol dose could be decreased when iPTH was ≤ 300 pg/mL and Ca × P was >55 mg^2^/dl^2^. The doses of Ca-based and other phosphate binders could be adjusted throughout the study. Ca-based phosphate binders could be increased when the serum Ca was <8.4 mg/dL or the patient had symptoms of hypocalcemia. Cinacalcet was given in a fixed low dose (30 mg/day) during the whole study period, and was withdrawn if the serum iPTH was persistently low (iPTH ≤ 300 pg/mL) for 4 weeks after the reduction of calcitriol or serum calcium <8.4 mg/dl or the patient had symptoms of hypocalcemia. All patients used low dialysate Ca 2.5 mEq/L and adjusted according to serum Ca levels throughout the study. A baseline visit was performed just before the start of the study, and further study visits were performed at 4, 8, 12, 16, 20 and 24 weeks after the medication. Serum biochemical parameters and bone turnover markers were assessed at baseline and during follow-up. The femoral neck (FN) and lumbar spine (LS) bone mineral density (BMD) were determined by dual X-ray absorptiometry (DXA) before and at the end of the study period. Hospital records were obtained and examined by two practicing nephrologists.

### 2.4. Serum Biochemical and Bone Metabolism Parameters

Blood samples were collected and serum was separated within 1 h of collection and immediately frozen until analysis. Serum levels of iPTH, 25(OH)D_3_, phosphorus, calcium and other bone metabolism parameters were determined. Serum iPTH levels were measured in an immunoradiometric assay (Nichols Institute Diagnostics, San Juan Capistrano, CA, USA). Serum 25(OH)D_3_ was determined by enzyme-linked immunosorbent assay (ELISA) according to the manufacturer’s instructions (Immundiagnostik AG, Bensheim, Germany). Serum bone specific alkaline phosphatase (BAP), a marker of bone formation was determined by ELISA (Quidel, Inc., San Diego, CA, USA); whereas tartrate-resistant acid phosphatase (TRACP)-5b, a marker of bone resorption was measured by ELISA (Quidel^®^, Tecomedical Group, Sissach, Switzerland). The doses of calcitriol, cinacalcet and phosphate binders were recorded at each visit. Adverse events were collected from patients’ reports and in response to non-directed questioning at each study visit.

### 2.5. Objectives and Outcomes and Measures

Our study used the Kidney Disease Outcomes Quality Initiative (K/DOQI) [30,31] targeted bone metabolism levels; serum Ca 8.4–9.5 mg/dL, P 3.5–5.5 mg/dL, iPTH 150–300 pg/mL. Interpretation of DXA scans using lumbar spine BMD was not precise in our patients since it might have been interfered with by aorta calcification and degenerative joint diseases. Thus, we used 10% improvement in femoral BMD instead of lumbar spine BMD as our outcome.

Primary Outcome: The primary outcome measure was serum iPTH ≤ 300 pg/mL. 

Secondary Outcome: The secondary outcome measures were serum 25-(OH)D_3_ ≥ 30 ng/mL and 10% improvement in femoral neck BMD from baseline.

### 2.6. Data Collection and Statistical Analysis

The results were expressed as mean ± standard deviation or median (interquartile range). Parametric or non-parametric tests were used for analysis; for paired data, the Student t or Wilcoxon tests, respectively, and for between-group comparisons, the Student t, one-way ANOVA or Mann–Whitney U tests were used. Unilateral correlation analysis was performed using Pearson (r) or Spearman correlation (rs), as appropriate. All the tests were two-sided, and *p* < 0.05 was considered statistically significant. Statistica (Version 11, Stat Soft, Inc., 2300 East 14th Street, Tulsa, OK 74104, USA) was used for calculations.

## 3. Results

### 3.1. Patient Recruitment and Analysis Sets

Table 1 shows the demographic characteristics of the study patients. All the patients were under HD with severe SHPT (serum iIPTH > 1000 pg/mL) or persistently high SHPT (serum iIPTH ≥ 600 pg/mL) even with 3 months of calcitriol treatment. Dialysis vintage, body mass index (BMI), underlying etiologies of ESRD and prior calcitriol usage were not significantly differing between two groups. All patients had high serum iPTH levels [CCC, 1034 ± 270 pg/mL vs. CCP, 1016 ± 252 pg/mL] and low 25(OH)D_3_ levels [CCC, 18.2 ± 8.4 ng/mL vs. CCP, 19.2 ± 7.4 ng/mL]. Serum albumin, ALP, cCa and P levels were not significantly different.

### 3.2. Changes in Serum Intact Parathyroid Hormone (iPTH) Levels during the Study Period

**Figure 2:**
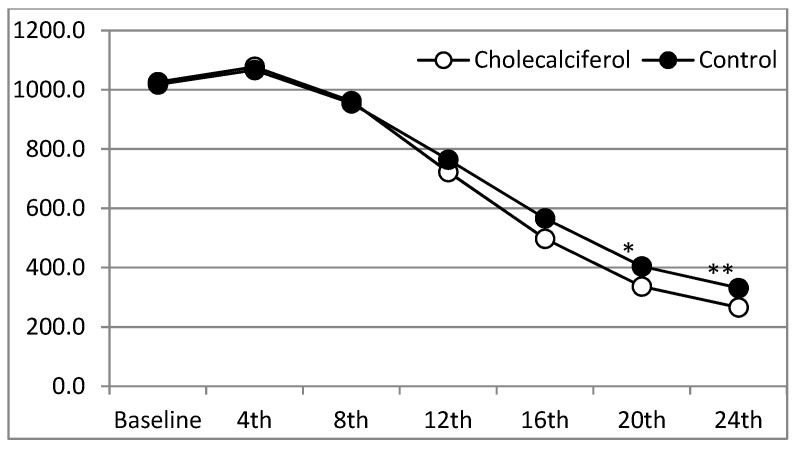
Changes in serum intact parathyroid hormone (iPTH) levels from baseline in two groups under study.

At 24 weeks, an average target iPTH level (≤300 pg/mL) was achieved in the CCC group but not in the CCP group (Table 2). The serum iPTH levels were significantly lower in the CCC group compared to the CCP group from the 20th week of the study, 336.4 ± 124.1 pg/mL in the CCC group vs. 404.4 ± 107.0 pg/mL in the CCP group (*p* = 0.034) at 20th week (Table 3 & Figure 2). The median changes in iPTH levels from baseline were significantly different as early as the 16th week of the study, −528.5 ± 148.1 pg/mL in the CCC group vs. −451.6 ± 116.0 pg/mL in the CCP group (*p* = 0.036) (Table 3). In subgroup analysis of the patients with severe vitamin D deficiency (25(OH)D3 < 12.5 ng/mL), serum iPTH levels were significantly different between the two groups at the 12th week, 486.5 ± 203.9 pg/mL in the CCC group vs. 841.5 ± 209.1 pg/mL in the CCP group (*p* = 0.024) (Table 4). The target iPTH level (≤300 pg/mL) was achieved from the 20th week in the CCC group compared with the CCP group in this subgroup of patients (Table 4). The mean dose of calcitriol use during the course of treatment was reduced progressively in the CCC group to control serum iPTH levels (Table 5). Dialysate calcium and doses of phosphate binder use did not differ significantly between the groups (data not shown). We further found that 11.1%, 29.6%, 55.6% and 81.5% of the study CCC group and 7.1%, 10.7%, 14.3% and 25% of the control CCP group achieved the target iPTH level at the 12th, 16th, 18th, 20th and 24th weeks, respectively (Table 6). Significantly more patients in the study group achieved the target level from the 20th week of the study (15/27 vs. 4/28, *p* = 0.046 at 20th wk; 22/27 vs. 7/28, *p* = 0.033 at 24th wk (chi-square test)) (Table 6). Most of our patients tolerated 30 mg of cinacalcet and none of the patients had hypocalcemia (cCa < 8.4 mg/dL) during the study period.

### 3.3. Changes in Serum 25(OH)D3 Levels during the Study Period

In the CCC group, 25(OH)D_3_ levels increased significantly from 18.2 ± 8.4 to 37.4 ± 9.6 (*p* < 0.01) at the end of the study. However, in the CCP group, 25(OH)D_3_ levels did not change significantly (from 19.2 ± 7.4 to 23.4 ± 7.5 (*p* = 0.46)) (Table 2). A significant number of patients in the CCC group achieved target 25(OH)D_3_ level (≥30 ng/dL) as early as the 12th week compared to the CCP group (21/27 (78%) vs. 2/28 (7%), *p* = 0.001 at 12th week); and nearly 89% of the CCC group vs only 10.7% in the CCP group achieved the target level at the end of the study (24/27 vs. 3/28, *p* = 0.001 (chi-square test)) (Table 6).

### 3.4. Changes in Serum Bone Turnover Markers and Bone Mineral Densities

Serum BAP and TRACP-5b levels were not significantly different between the groups (Table 2). No obvious changes in serum Alk-P, cCa and P were noted. Although no significant differences in femoral neck and lumbar spine BMD were noted between the two groups (Table 7), the number of patients who had 10% increase in FN BMD was insignificantly higher in the CCC group ((13/27) 40%) compared with the CCP group ((5/28) 6.7%) (Table 6).

## 4. Discussion

Our results demonstrated that more than 80% of patients with severe SHPT achieved the primary target serum iPTH ≤300 pg/ml level with cholecalciferol supplementation. Nearly 90% of patients achieved the target serum 25-(OH)D_3_ (≥30 ng/mL) with high-dose cholecalciferol supplementation (5000 IU/day). About 40% of patients receiving cholecalciferol achieved a 10% increase in FN BMD by the end of the study. To our knowledge, this is the first study to reveal the beneficial effects of cholecalciferol supplementation in further lowering iPTH and bone density improvement when combined with cinacalcet and calcitriol among severe SHPT patients.

As previously described, cinacalcet significantly increases the oxyphil cells (OC) and chief cells (CC) OC/CC ratio (approx. increase 3.42 times) [24], further increases local calcitriol production [26], and regulates local PTH secretion in a paracrine/autocrine manner. Thus, the need for local 25(OH)D_3_ substrate increases with cinacalcet treatment. We proved in our study that addition of nutritional vitamin D (cholecalciferol) improves the 25(OH)D_3_ requirement by cinacalcet treatment, and further inhibits PTH secretion among those patients with severe SHPT when used in combination with cinacalcet/active vitamin D analogs. About 55% of the patients achieved the iPTH target (iPTH ≤ 300 pg/mL) at the 20th week and almost 81% of patients achieved that target after the 24th week of cholecalciferol supplementation. This effect was also seen among those patients with severely deficient vitamin D levels (serum 25(OH)D_3_ < 12.5 ng/mL). Furthermore, a higher percentage of patients achieved the primary endpoint in the CCC group, irrespective of the prior use of active vitamin D, calcitriol.

Although the CCP group also significantly improved iPTH levels, the target iPTH level was not achieved in most patients by the end of the study. Previous studies also proved that cinacalcet therapy improved the targets of bone metabolism (PTH, Ca, P, and the Ca-P product (Ca-P)) when used alone [32] or in combination of low-dose active vitamin D analogs [7]. However, all these studies used titrated doses of cinacalcet (30–180 mg per day) according to biochemical measures. We used a single daily dose of cinacalcet (30 mg per day) throughout our study, since the drug is not covered by health insurance in Taiwan and patients took the drug by self-payment. We also found that lower doses of calcitriol were needed among our study group with time (Table 5). Most of the CCC group patients achieved KDOQI targets [33] (serum PTH 150–300 pg/mL, Ca 8.4–9.5 mg/dL, P 3.5–5.5 mg/dL, CaxP product < 55 mg^2^/dL^2^) and none had hypocalcemia. Since hypocalcemia is the major event prohibiting the use of cinacalcet, and hypercalcemia is a major complication of active vitamin D analogs, the supplementation of nutritional vitamin D avoids both hypo- and hypercalcemic events in such combination therapy.

We found that up to 70% of CCC patients achieved the target 25(OH)D_3_ level (≥30 ng/mL) from the 12th week of supplementation and nearly 90% had the target level by the end of the study. However, no such improvement was seen in the CCP group. Clinical studies reported that the combined use of nutritional vitamin D might reduce the active vitamin D doses [3,34] and improve adverse effects. Accordingly, we found our CCC group patients used progressively lower doses of calcitriol compared to the CCP group. Studies regarding the effective dosage of vitamin D supplementation are still controversial [35,36]. Our previous study used low-dose cholecalciferol (5000 IU/week) and about 60% of patients achieved the target 25(OH)D_3_ level (≥30 ng/dL) at 16 weeks [10]. Growing evidence shows that active vitamin D analogs may aggravate 25(OH)D_3_ deficiency because of the feedback inhibition of hepatic 1α-OHase and 25α-OHase [37,38] and induction of the 24-OHase enzyme [39]. Thus, we used quite a high dose of cholecalciferol (5000 IU/day) in combination with calcitriol in our study. We closely monitored 25(OH)D_3_ levels to avoid toxicity, and all of our patients tolerate high-dose cholecalciferol supplementation (5000 IU/day) well.

The next major observation of our study was that about 40% in the CCC group gained >10% improvement in femoral BMD compared with the CCP group (~7%). Other clinical studies also revealed that calcimimetic agents reduced the elevated bone formation rate/tissue area with improvement in high turnover bone disorders [40,41]. In a recent Indian study among CKD patients, researchers found a significant reduction in iPTH and bone turnover markers, bone-specific alkaline phosphatase (BAP) and serum C-terminal cross-linked collagen type I telopeptides (CTX-1) with cholecalciferol supplementation [42]. Previous studies [43,44] also proved that cinacalcet treatment improves the BMD of the FN without affecting the LS BMD. How and whether the combination of these agents interacts to improve bone parameters needs to be explored in future studies.

Our study has several limitations. Firstly, we cannot apply our findings or draw definitive conclusions in the general HD population due to the relatively small sample size; however, the results were indeed promising and clinically important. We used fixed low doses of cinacalcet in both study and control arms, which needed to be followed longer for efficiency. Furthermore, we excluded patients with severe malnutrition and inflammatory or infectious disorders who might have benefited the most from the intervention due to vitamin D deficiency. However, our cohort had significantly low vitamin D levels in both arms of the study. Next, we did not determine the morbidity or mortality benefits of combination therapy. Although we performed some questionnaires on various bone fractures, the results were inconsistent and non-significant due to shorter follow-up duration. The optimal serum 25(OH)D_3_ target level in dialysis patients remains unknown; however, we supposed some additive PTH lowering effects with 25(OH)D_3_ ≥ 30 ng/dL. Although no toxicity levels and signs were noted in our patients, the beneficial role and possible side effects of high-dose cholecalciferol in dialysis patients still requires a multifaceted long-term approach and needs further study in larger clinical trials.

## 5. Conclusions

Our findings provide in vivo evidence that 25(OH)D_3_ repletion with high-dose cholecalciferol acts additively with low-dose cinacalcet and active vitamin D analogs to gain K/DOQI targets of bone metabolism markers and improve femoral neck bone mineral density in dialysis patients. We recommend that the addition of cholecalciferol is more favorable than the titration of cinacalcet and calcitriol doses among severe SHPT patients.

## Figures and Tables

**Table 1:** Demographic characteristics and baseline data of the two groups under study.

Characteristics	CCC (*n* = 27)	CCP (*n* = 28)
Age, mean ± SD (years)	66.2 ± 12.8	65.6 ± 13.4
Male, *n* (%)	16 (59%)	14 (50%)
BMI (kg/m^2^)	21.94 ± 3.48	22.32 ± 4.14
HD vintage (months)	52.8 ± 28.5	54.5 ± 29.6
GN, *n* (%)	4 (15%)	6 (21%)
DM, *n* (%)	13 (48%)	12 (43%)
HTN, *n* (%)	5 (19%)	4 (14%)
Others, *n* (%)	5 (19%)	6 (21%)
Prior calcitriol usage, *n* (%)	25 (93%)	24 (86%)
iPTH (pg/mL)	1026 ± 266	1018 ± 261
25(OH)D_3_ (ng/mL)	18.2 ± 8.4	19.2 ± 7.4
Albumin (g/dL)	3.62 ± 0.41	3.64 ± 0.38
Alkaline phosphatase (U/L)	301.2 ± 84.3	292.8 ± 92.4
cCa (mg/dL)	10.4 ± 0.61	10.2 ± 0.65
P (mg/dL)	5.18 ± 0.84	5.24 ± 0.76

Data are expressed as the mean ± SD, *n* (%), or median (interquartile range). SD: standard deviation; BMI, body mass index; HD, hemodialysis; GN, primary glomerulonephritis; DM, diabetes mellitus; HTN, hypertension; Others, vascular or ischemic nephropathy and tubulointerstitial nephritis; iPTH, parathyroid hormone; 25(OH)D_3_, 25-Hydroxyvitamin D; cCa, albumin-corrected calcium; P, phosphorus. CCC: Cinacalcet + Calcitriol + Cholecalciferol; CCP: Cinacalcet + Calcitriol + Placebo.

**Table 2:** Serum biochemistry parameters change among the two groups under study.

Parameters	CCC (*n* = 27)			CCP (*n* = 28)		
	Before	After	*p* Value	Before	After	*p* Value
iPTH (pg/mL)	1026 ± 266	265.8 ± 47.0	<0.01	1018 ± 261	326.1 ± 77.3	<0.01
25(OH)D_3_ (ng/mL)	18.2 ± 8.4	37.4 ± 9.6	<0.01	19.2 ± 7.4	23.4 ± 7.5	0.46
Alk-P (U/L)	301.2 ± 84.3	121.4 ± 48.6	<0.01	292.8 ± 92.4	148.4 ± 58.6	<0.01
BAP (U/L)	42.2 ± 8.4	23.6 ± 7.2	<0.01	44.5 ± 9.2	28.62 ± 6.8	<0.01
TRACP-5b (U/L)	7.8 ± 3.8	2.4 ± 1.6	<0.01	7.6 ± 3.7	3.2 ± 1.8	<0.01
Albumin	3.62 ± 0.41	3.72 ± 0.39	0.08	3.64 ± 0.38	3.86 ± 0.41	0.24
cCa (mg/dL)	10.4 ± 0.61	9.16 ± 0.54	0.26	10.2 ± 0.65	9.24 ± 0.61	0.41
P (mg/dL)	5.18 ± 0.84	5.01 ± 0.54	0.49	5.24 ± 0.76	5.14 ± 0.62	0.77

iPTH, parathyroid hormone; 25(OH)D3, 25-Hydroxyvitamin D; Alk-P, alkaline phosphatase; BAP, bone alkaline phosphatase; TRACP-5b, tartrate-resistant acid phosphatase 5b; cCa, albumin-corrected calcium; P, phosphorus.

**Table 3:** Serum iPTH levels and mean iPTH change from baseline in the two groups under study.

Week	CCC (*n* = 27)	CCP (*n* = 28)	*p* Value *	CCC (*n* = 27)	CCP (*n* = 28)	*p* Value *
	iPTH (pg/mL)	iPTH (pg/mL)		△iPTH (pg/mL)	△iPTH (pg/mL)	
Baseline	1026.1 ± 266.1	1018.0 ± 260.5	0.909	-	-	-
4th week	1077.9 ± 274.8	1066.7 ± 274.2	0.881	51.8 ± 159.1	49.0 ± 116.2	0.942
8th week	961.7 ± 243.0	954.4 ± 236.6	0.910	−64.2 ± 39.7	−63.2 ± 39.7	0.925
12th week	722.5 ± 247.3	764.5 ± 191.0	0.483	−303.5 ± 151.2	−253.2 ± 75.9	0.123
16th week	497.6 ± 182.5	566.1 ± 153.7	0.137	−528.5 ± 148.1	−451.6± 116.0	0.036
20th week	336.4 ± 124.1	404.4 ± 107.0	0.034	−689.6 ± 172.9	−613.3 ± 161.0	0.096
24th week	265.8 ± 47.0	326.1 ± 77.3	0.001	−760.3 ± 257.3	−686.8 ± 212.0	0.252

* *t*-test.

**Table 4:** Serum iPTH levels changes in patients with baseline 25(OH)D3 < 12.5 ng/mL.

Week	CCC (*n* = 6)	CCP (*n* = 5)	*p* Value
Baseline	1034.6 ± 282.4	1124.2 ± 284.6	0.612
4th week	1038.4 ± 284.8	1106.7 ± 274.2	0.697
8th week	864.3 ± 262.1	965.4 ± 266.4	0.543
12th week	486.5 ± 203.9	841.5 ± 209.1	0.024
16th week	354.2 ± 153.5	642.2 ± 193.7	0.022
20th week	265.7 ± 108.3	482.3 ± 168.8	0.029
24th week	225.5 ± 34.1	384.6 ± 136.4	0.021

**Table 5:** Mean dose of intravenous calcitriol (μg/week) during the course of treatment.

Week	CCC (*n* = 27)	CCP (*n* = 28)
Baseline	3 μg/week	3 μg/week
4th week	3 μg/week	3 μg/week
8th week	3 μg/week	3 μg/week
12th week	2.67 μg/week	3 μg/week
16th week	2.22 μg/week	2.79 μg/week
20th week	1.44 μg/week	2.57 μg/week
24th week	0.56 μg/week	2.25 μg/week

**Table 6:** Number of study patients achieving primary and secondary outcomes in the two groups under study.

**Primary Outcome**	**12th Week**	**16th Week**	**20th Week**	**24th Week**
iPTH ≤ 300 pg/mL				
CCC (*n* = 27)	3/27 (11.1%)	8/27 (29.6%)	15/27 (55.6%)	22/27 (81.5%)
CCP (*n* = 28)	2/28 (7.1%)	3/28 (10.7%)	4/28 (14.3%)	7/28 (25%)
	*p* = 1.000	*p* = 0.270	*p* = 0.046	*p* = 0.033
**Secondary Outcome**	**12th Week**	**16th Week**	**20th Week**	**24th Week**
25(OH)D_3_ ≥ 30 ng/dL				
CCC (*n* = 27)	21/27 (77.8%)			24/27 (88.9%)
CCP (*n* = 28)	2/28 (7.1%)			3/28 (10.7%)
	*p* = 0.001			*p* = 0.001
↑ FN-BMD > 10%				
CCC (*n* = 27)				13/27 (40%)
CCP (*n* = 28)				5/28 (6.7%)
				*p* = 0.150

**Table 7:** Bone mineral density (BMD) changes after 24 weeks of treatment in the two groups under study.

Parameters	CCC (*n* = 27)			CCP (*n* = 28)		
	Before	After	*p* Value	Before	After	*p* Value
FN-BMD (g/cm^2^)	0.57 ± 0.04	0.67 ± 0.07	<0.05	0.58 ± 0.05	0.62 ± 0.06	<0.05
FN-T score	−2.1 ± 0.5	−1.2 ± 0.4	<0.05	−2.2 ± 0.6	−1.8 ± 0.5	<0.05
LS-BMD (g/cm^2^)	0.91 ± 0.09	0.96 ± 0.10	<0.05	0.89 ± 0.07	0.94 ± 0.08	<0.05
LS-T score	−0.7 ± 0.8	−0.6 ± 0.7	0.627	−0.8 ± 0.7	−0.7 ± 0.6	0.568

Data are expressed as mean ± standard error.
